# A Case of Renal Calculus with Operation

**Published:** 1904-07

**Authors:** Jno. B. Thomas

**Affiliations:** Abilene, Texas


For Texas Medical Journal.

### A Case of Renal Calculus With Operation.

JNO. B. THOMAS, M. D., ABILENE, TEXAS.

*Patient:* W. N. E.; male, white, blonde type, age 30; married eight years, two children. Born at McKinney, Texas. Been living about and in Abilene for twenty years. Social and family histories negative. Never used stimulants or narcotics of any kind. Father, mother and other members of the family all in good health. No history of any hereditary tendency whatever. Had ordinary disease of childhood. Typhoid fever when twelve years old and pneumonia two years ago. Good recoveries in each instance. La grippe six months ago. Several weeks regaining former strength.

*History:* Since four years of age has complained of pain in right side in region of kidney. When twelve years old had first attack of severe suffering. At about that time he would go perhaps two weeks without any distinct pain, but at all times conscious of a kind of soreness in right side, the attacks of an agonized character only coming on every few weeks or months. Avoidance of more than moderate exertion and deliberateness of exercise had a tendency to prolong the intervals between the severest attacks. There never was any radiation of pain, it always being in the same place and often of a sickening character, sometimes sharp and almost intolerable.

When patient came under observation (February 1, 1904), a correct diagnosis had never been suggested to him until six months ago. Some said he had too much lime in his bladder, and once he was told that his trouble was neuralgia of the kidney. During all this time no alteration in the character of the urine was ever noticed by the patient, until attention was called to it after examination. Was never conscious that blood or pus was being passed, and never any symptom of cystitis.

*Examination:* When first seen patient's weight was 149 pounds. General condition, fairly good. Slightly anemic. Expression of face gave evidence of the anxiety and suffering to which he had been subjected for years. The continuous pain in side was somewhat worse than formerly, and agonizing attack perhaps of greater violence and more frequent. On firm pressure over kidney only the slightest soreness could be elicited. No enlargement visible.

The urine was examined and found to contain one-fourth to onethird its volume of pus, and occasionally a very few red blood corpuscles, never, however, in sufficient number to give any physical coloration to the specimen. Though repeated microscopic examinations were made, no crystals of any description or casts were ever found. Yet the latter may have been overlooked in the midst of the myriads of pus cells. The specific gravity was normal; reaction, acid. Small amount of mucus and only a few epithelial cells. Reaction for albumen, positive, but thought due to the presence of pus and some blood.

Believing we had to contend with renal calculus with infection, before suggesting any method of treatment, decided to determine condition of the opposite kidney. Avoiding the possibility of infecting the presumably healthy organ by a cystoscopic examination and ureteral catheterization, the urine from the two kidneys was separated by means of Harris' urine segregator. This was a beautiful demonstration of the usefulness of this instrument. The bladder having been thoroughly irrigated with sterile water, the urine was allowed to accumulate and drain away into the receptacles. The one receiving the secretion of the right kidney contained turbid urine from admixture with considerable quantity of pus; the other received urine from the left kidney, perfectly normal in appearance. No albumen, and on microscopic examination none of the elements of disease.

*Operation:* March 15th. The vertical limb of Konig's lumbar incision, slightly oblique, about three inches in length, was employed. When kidney was reached organ was brought slightly into the wound. Perirenal tissue separated and surface explored as far as hilum. The kidney was about normal in size, and on palpation nothing suggestive of stone could be found. The renal substance having already been found to be quite friable, a sharp-pointed hemostatic forceps was pushed from convexity easily into pelvis of kidney. Along this tract the finger was introduced into a cavity about the size of a hen's egg, communicating freely with pelvis. Running in different directions throughout this cavity were a number of septa. Lying loosely in the cavity was a calculus, rather smooth in outline, weighing three grams, one-half by one inch in diameter. The stone was removed; the septa broken down. The cavity and orifice of the ureter were thoroughly explored. Into the latter, tip of the finger could be readily introduced. Its mucous membrane was smooth, and, to the touch, in a healthy condition. The wound was irrigated with hot, sterile, saline solution. A

drainage tube was inserted to opening of ureter and packed round about with iodoform gauze. The hemorrhage which up to this time had been somewhat profuse, very quickly checked.

Not a single ligature was used during the entire operation. External wound was closed with silk-worm gut sutures up to the space where drainage emerged.

The shock was only moderately severe. High rectal injections of saline solution were given every three hours during the first twenty-four following the operation. Stimulants were given as indicated. Temperature slightly subnormal at first, soon reached normal and continued so. Pulse, after first twenty-four hours, was never above 100. On fifth day both temperature and pulse became normal and remained so throughout convalescence. During first twenty-four hours following operation two pints of bloody urine passed, showing that not only were kidneys functionating satisfactorily, but that there was no obstruction to this avenue of drainage from affected kidney. The urinary secretion since operation has ranged in amount from two to four pints per day. Diet for several days consisted mainly of milk with a rigorous restriction of albuminous food. Water has been taken in systematic abundance since relief of nausea, second day after operation.

Drainage through lumbar wound was maintained until all signs of infection disappeared and urine became perfectly healthy and sweet. Tube was then withdrawn from kidney and renal tract coaxed to close up, which it very promptly did. When deeper end of the fistulous opening closed, tube was withdrawn completely. Now, ten weeks since operation, patient is apparently entirely well, without a symptom of renal or any other disease. Weight, 148 pounds.

This case has been particularly interesting to the writer, not only because of its having been dealt with so early in his experience, but because of the long undisturbed existence of a stone in the kidney; and I would suggest that the remarkable duration without any serious complication was doubtless due to the perfect system of drainage supplied by nature.

---

"Wives and daughters all remind us
We must make our little pile,—
And, departing, leave behind us
Cash, that they may live in style."

—*Tid-Bits.*

Next!

